# Cohen Syndrome Patient iPSC-Derived Neurospheres and Forebrain-Like Glutamatergic Neurons Reveal Reduced Proliferation of Neural Progenitor Cells and Altered Expression of Synapse Genes

**DOI:** 10.3390/jcm9061886

**Published:** 2020-06-16

**Authors:** You-Kyung Lee, Su-Kyeong Hwang, Soo-Kyung Lee, Jung-eun Yang, Ji-Hye Kwak, Hyunhyo Seo, Hyunjun Ahn, Yong-Seok Lee, Janghwan Kim, Chae-Seok Lim, Bong-Kiun Kaang, Jae-Hyung Lee, Jin-A Lee, Kyungmin Lee

**Affiliations:** 1 Department of Biotechnology and Biological Sciences, Hannam University, Daejeon 34430, Korea; alskgogo77@gmail.com (Y.-K.L.); cw02374@gmail.com (S.-K.L.); 2 Department of Pediatrics, School of Medicine, Kyungpook National University, Daegu 41944, Korea; neurobaby79@gmail.com; 3 Department of Biological Sciences, College of Natural Sciences, Seoul National University, Seoul 08826, Korea; yje1001@gmail.com (J.-e.Y.); kaang@snu.ac.kr (B.-K.K.); 4 Laboratory for Behavioral Neural Circuitry and Physiology, Department of Anatomy, Brain Science & Engineering Institute, School of Medicine, Kyungpook National University, Daegu 41944, Korea; jhkwak@knu.ac.kr (J.-H.K.); hseo@knu.ac.kr (H.S.); 5 Stem Cell Convergence Research Center, Korea Research Institute of Bioscience and Biotechnology (KRIBB), Daejeon 34141, Korea; handisilver@gmail.com (H.A.); janghwan546@gmail.com (J.K.); 6 Department of Functional Genomics, KRIBB School of Bioscience, University of Science and Technology, Daejeon 34113, Korea; 7 Department of Physiology, Biomedical Sciences, Neuroscience Research Institute, Seoul National University College of Medicine, Seoul 03080, Korea; yongseok7@snu.ac.kr; 8 Department of Pharmacology, Wonkwang University School of Medicine, Iksan 54538, Korea; cslimwk1@wku.ac.kr; 9 Department of Life and Nanopharmaceutical Sciences, Department of Oral Microbiology, School of Dentistry, Kyung Hee University, Seoul 02447, Korea

**Keywords:** Cohen syndrome, *VPS13B*, induced pluripotent stem cells (iPSCs), neurosphere, transcriptomic analysis

## Abstract

Cohen syndrome (CS), a rare autosomal recessive disorder, has been associated with genetic mutations in the *VPS13B* gene, which regulates vesicle-mediated protein sorting and transport. However, the cellular mechanism underlying CS pathogenesis in patient-derived human neurons remains unknown. We identified a novel compound heterozygous mutation, due to homozygous variation of biparental origin and heterozygous variation inherited from the father, in the *VPS13B* gene in a 20-month-old female patient. To understand the cellular pathogenic mechanisms, we generated induced pluripotent stem cells (iPSCs) from the fibroblasts of the CS patient. The iPSCs were differentiated into forebrain-like functional glutamatergic neurons or neurospheres. Functional annotation from transcriptomic analysis using CS iPSC-derived neurons revealed that synapse-related functions were enriched among the upregulated and downregulated genes in the CS neurons, whereas processes associated with neurodevelopment were enriched in the downregulated genes. The developing CS neurospheres were small in size compared to control neurospheres, likely due to the reduced proliferation of SOX2-positive neural stem cells. Moreover, the number of SV2B-positive puncta and spine-like structures was significantly reduced in the CS neurons, suggesting synaptic dysfunction. Taking these findings together, for the first time, we report a potential cellular pathogenic mechanism which reveals the alteration of neurodevelopment-related genes and the dysregulation of synaptic function in the human induced neurons differentiated from iPSCs and neurospheres of a CS patient.

## 1. Introduction

Cohen syndrome (CS) is a rare autosomal recessive disorder characterized by intellectual disability, postnatal microcephaly, facial dysmorphism, and/or motor abnormalities, with significant variability in the spectrum of its clinical features [1,2]. CS is associated with mutations in the *VPS13B* (also known as *COH1*) gene, which encodes a protein of 4022 amino acids (up to 62 exons) and includes various splicing isoforms. The human VPS13 (hVPS13) protein family has four widely expressed forms: hVPS13A, hVPS13B, hVPS13C, and hVPS13D. Interestingly, mutations or perturbations in *hVPS13* genes are associated with various human diseases, including neurodegenerative diseases, neurological disorders, cancers, and diabetes [3]. Among hVPS13 family proteins, hVPS13B, which is associated with intellectual disability and autism, regulates the morphology of the Golgi complex and the glycosylation of proteins [4]. In post-mitotic rodent neurons, VPS13B has been reported to regulate neurogenesis via its interaction with Rab6 GTPase [5]. A recent study showed that VPS13B also functions as a tethering factor involved in the transport from early endosomes to recycling endosomes by binding to syntaxin13/syntaxin6, as well as Rab14, Rab6, and Ptdlns(3)p [6]. Moreover, according to the Human Mutation Database [7], the total number of mutations of the *VPS13B* gene is the highest of all the paralogs of human *VPS13* genes, including point mutations, small rearrangements, or gross rearrangements. Intriguingly, although homozygous or compound heterozygous mutations in *VPS13B* are identified in most CS patients, only one heterozygous mutation is detected in about 20%–30% of patients, whereas no mutations are identified in 12% of patients, indicating that other genetic mutations and environmental factors are also related to CS pathogenesis [8]. For these complex cases, the underlying cellar mechanism that causes each case of CS remains largely unknown. 

In a recent report, *VPS13B* knockout mice failed to form an acrosome, and mice with the deletion of *VPS13B* exon 2 had impaired motor activity and spatial learning, suggesting that *VPS13B* mutant mice are a useful model of CS pathogenesis in vivo [9,10]. However, there are several limitations to investigating the pathophysiological mechanisms of CS using these rodent models, due to either early lethality or limited face validity. Therefore, induced pluripotent stem cell (iPSC) technology using patient-derived cells may provide a powerful compensatory tool for modeling the cellular pathogenesis of CS.

Patient-derived iPSC models can be used to study the disease mechanisms of neurological disorders involving complex genetic mutations, such as autism, non-familial cases of human diseases, or rare human diseases [11]. In addition, three-dimensional (3-D) neurospheres or region-specific brain organoids which are differentiated from human iPSCs may be the best models for human early brain development [12], such as microcephaly, which is one of the clinical phenotypes observed in CS patients [2]. However, so far, to our knowledge, there is no human patient-derived neuronal and neurosphere model to characterize the cellular pathogenesis of CS using patient-specific, personalized induced pluripotent stem cells (iPSCs).

In this study, to establish a human cellular disease model of CS, we generated personalized iPSCs from the skin fibroblasts of an individual CS patient with two novel compound point mutations in the exonic region of *VPS13B*. Then, we sought to clarify its cellular pathogenesis in forebrain-like glutamatergic neurons and neurospheres. Interestingly, the CS patient-derived neurons had significantly altered expression in neurodevelopment, cell proliferation, and synaptic function-related genes. In addition, the 3-D neurospheres which were differentiated from CS patient iPSCs were abnormally small, suggesting a dysregulated proliferation of neural progenitor cells (NPCs) which were differentiated from the CS patient. Therefore, our CS iPSC-derived forebrain-like neurons or smaller neurospheres could provide a novel cellular pathogenic mechanism of CS which reveals a reduced cell proliferation of NPCs or altered expression of synapse-associated genes.

## 2. Materials and Methods

### 2.1. Genetic Analysis of the CS Patient 

The subject was a 20-month-old female patient who was brought in for the evaluation of multiple congenital anomalies and global developmental delay. Detailed clinical and family histories were obtained. Genetic analysis included chromosomal microarray, whole-exome sequencing, and Sanger sequencing. Genomic DNA was extracted from ethylenediaminetetraacetic acid (EDTA)-treated whole blood samples using a QIAamp DNA Blood Mini kit (ID: 51106; Qiagen, Hilden, Germany). DNA quality and quantity were assessed using a Qubit Fluorometer (Invitrogen, Carlsbad, CA, USA) and a Quant-iT BR assay kit (Q32850; Invitrogen, USA). The institutional review board of Kyungpook National University Hospital approved the protocol, and informed consent was obtained for the genetic analysis and the use of the results for diagnosis and research purposes from the patient’s legal guardian (IRB no. KNUH 2016-06-011 and HANAM IRB13K (13-12)). Whole-exome sequencing was performed and targeted exonic regions were captured with the Agilent SureSelect XT Human All Exon v5 kit (ID: 5190-6209, Agilent Technologies, Santa Clara, CA, USA) on the Illumina HiSeq-2000 (Illumina, San Diego, CA, USA) platform. Sanger sequencing was performed in order to confirm the suspected pathogenic mutations identified using whole-exome sequencing and to determine whether other family members had the same mutation. The target site of the variant and the flanking DNA sequences from the patient and each family member were amplified with forward and reverse primers (primer sequences: for the identification of c.1239T>G mutation, forward primer, GGCGAGGAAGACTTTGTT, reverse primer, TATCTCACATCTACTGAATGC; for the identification of c.10333G>A mutation, forward primer, GCCTGGCAGTGTTTGATGA, reverse primer, GTTGGATATGAGGAGACTCTGC). The amplified products were directly sequenced using an automated DNA sequencer (ABI3130; Applied Biosystems, Foster City, CA, USA) using a Big-Dye Terminator Cycle Sequencing kit version 3.1. The primer sequences are available upon request.

### 2.2. Cell Culture and Generation of iPSCs

CS iPSC and control iPSC lines were generated using a non-integrating induction method with episomal vectors from human skin fibroblasts obtained via skin biopsy, as described previously [13]. Briefly, on day 7 after electroporation, cells were reseeded onto a feeder layer of mouse embryonic fibroblasts (MEFs). iPSC lines were maintained using an embryonic stem cell (ESC) medium containing 20% knockout serum (10828-028, Gibco, Carlsbad, CA, USA), beta-mercaptoethanol (21985-023, Gibco, Carlsbad, CA, USA), 1× GlutaMAX (3500-061, Gibco, Carlsbad, CA, USA), 1× MEM-NEAA (1140-050, Gibco, USA), penicillin/streptomycin (15140-122, Gibco, Carlsbad, CA, USA), and DMEM-F12 (11320-033, Gibco, Carlsbad, CA, USA). After 1 month, iPSC-like colonies were picked and reseeded on new feeder cells. We changed the iPSC culture system from MEF feeder-dependent to the feeder-free culture system using the essential 8 medium (A1517001, Gibco, Carlsbad, CA, USA) at passage 5–7. To confirm the mutation in the VPS13B gene in the iPSCs, we performed a genomic PCR using specific primers (VPS13B sense (1)-5′-CCGGACTGCAAGACCAAAGA, antisense (1)-5′-TGGCTGGATCACCAGTTTCC; sense (2)-5′-CAACTGAGTGGAGTGATGCCA, antisense (2)-5′- GCTGGATCACCAGTTTCCGTA).

### 2.3. Rapid Neuronal Induction from iPSCs by Neurogenein2 (NGN2)

For glutamatergic neuronal induction, we used the neurogenin2 (NGN2)-mediated rapid neural induction protocol with some modifications [14]. On day 1, iPSCs were dissociated using accutase (AT104, Innovative cell Tech, San Diego, CA, USA) and plated on Matrigel (#354230, Coning, New York, NY, USA)-coated 6-well plates in mTeSR1 media (#ST85850, Stem cell technology, Vancouver, BC, Canada) containing 10-µM Y-27632 (#1254, Tocris, Bristol, UK). On day 2, rtTA+Ngn2 lentiviruses [14] were added to the cultures. On day 3, the culture medium was changed to an induction medium (N2/MEM-NEAA/DMEM-F12 plus penicillin/streptomycin) containing 10-µg/L human brain-derived neurotrophic factor (BDNF) (#450-02, Peprotech, Rocky Hill, NJ, USA), 10-µg/L human NT-3 (#450-03, Peprotech, Rocky Hill, NJ, USA), 0.2-mg/L mouse laminin (#L2020, Sigma, St. Louis, MO, USA), and 2-g/L doxycycline (#631311, Clontech, Kusatsu, Japan). On day 4, puromycin (1 mg/L) (#540411, Millipore, Burlington, MA, USA) was added in order to select the virus-infected cells for 24 h. On day 5, the culture medium was replaced with 1:1 mixed glial-conditioned medium and a Neurobasal/B27/GlutaMAX growth medium containing 10-µg/L human BDNF, 10-µg/L human NT-3, 2-g/L doxycycline, 2-g/L Ara-C (#c6645, Sigma, St. Louis, MO, USA), and 1-mg/L puromycin. As for the characterization of the synaptic structures (SV2B-positive puncta/spine structure), rapid neuronal induction efficiency, and electrophysiological analysis, mouse astrocytes (1.0 × 10^4^ cells/well) were plated on Matrigel-coated 24-well plates for preparing the co-culture. On days 4–8, 50% of the medium was replaced with 1:1 mixed glial cell-conditioned medium and a Neurobasal/B27/GlutaMAX growth medium containing 10-µg/L human BDNF, 10-µg/L human NT-3, 2-g/L doxycycline, 2-g/L Ara-C, and 1-mg/L puromycin every 2 days. Ara-C was added to inhibit the proliferation of cells after neural differentiation. On day 9, the cultures were washed with Dulbecco’s phosphate-buffered saline (DPBS) once and treated with trypsin for 10 min at 37 °C, and then the dissociated neurons were collected by centrifugation at 200*g* for 3 min. The neurons were plated (5 × 10^5^ cells/24 well) on the glia-plated coverslips (or on the coverslip without mice astrocytes cultures for RNA sequencing analysis or Western blot analysis) in 500-µL Neurobasal/B27/GlutaMAX growth medium containing 10-µg/L BDNF, 10-µg/L NT-3, and 2-g/L doxycycline. From day 8 onward, 50% of the medium was replaced with a Neurobasal/B27/GlutaMAX/fetal bovine serum (FBS) (2.5%) medium containing 10-µg/L BDNF, 10-µg/L NT-3, and 2-g/L doxycycline every 5–7 days.

### 2.4. Immunocytochemistry

To assess the expression of stem cell- or neuron-specific markers, we performed immunocytochemistry [15,16] using the antibodies of stem cell markers (Oct3/4, #sc-5279/Santa Cruz/USA/1:100; Nanog, #RCAB003P-F/Reprocell/USA/1:50; TRA-1-60, #09-0068/STEMGENT/USA/1:50 or #MAB4360/Millipore/USA/1:50; TRA-1-81, #09-0069/STEMGENT/USA/1:50 or #MAB4381/Millipore/USA/1:50) and neuronal markers (SV2B, #119102/Synaptic Systems/Germany/1:100; doublecortin (Dcx), #sc-271390/Santa Cruz/USA/1:200; vGLUT, #135303 Synaptic Systems/Germany/1:500; GAD67, #MAB5406/Millipore/USA/1:500; or MAP2, #AB5622-I Millipore/USA /1:500). Four weeks after rapid neuronal induction, the iPSCs or induced neurons were fixed with 4% paraformaldehyde for 15 min at room temperature and washed with 1× phosphate-buffered saline (PBS). For permeabilization, we used 0.1%–1% Triton X-100 for 15 min, and then the fixed cells were blocked with 3% bovine serum albumin (BSA) in PBS for 1 h at room temperature. Primary antibodies were incubated overnight at 4 °C. Alexa Fluor 488 (#715-545-150, #711-545-152/Jackson ImmunoResearch/USA/1:200) and Cy3-conjugated secondary antibodies (#715-165-150, #711-165-152/Jackson ImmunoResearch/USA/1:200) were incubated at room temperature for 90 min and were detected by confocal microscopy (ZEISS/Germany/LSM 880). We counted the number of SV2B-positive puncta on doublecortin (DCX)-positive neurites per 20 μm for quantification and the number of green fluorescence protein (GFP)-positive spine structure (indicated by the protrusion from dendrites) per 25 μm in control, CS1, or CS2 induced neurons [17]. For the immunostaining of the neurospheres, the cells were fixed with 4% formaldehyde and embedded in 30% sucrose. Next, 30-μm-thick sections were made using a cryostat (Leica, Germany). Then, the sections were attached onto the glass slide for immunostaining. After washing the slide with 1× PBS, we permeabilized the fixed organoids using 0.1% Triton X-100 for 15 min and blocked them with 5% goat serum and 0.1% Triton X-100 in PBS for 1 h at room temperature (RT). The primary antibodies diluted in the blocking solution were incubated overnight at 4 °C, and the secondary antibodies were incubated for 2 h at room temperature.

### 2.5. Reverse Transcription and Quantitative Real-Time (RT) PCR

For mRNA extraction, we used TRIzol and a total RNA extraction miniprep kit (#T2010S, NEB, USA). cDNA synthesis was performed using a Superscript III reverse transcription kit and a LunaScript RT Supermix kit (#E3010L, NEB, Ipswich, MA. USA). To validate the mRNA expression levels, semi- or real-time quantitative PCR was performed using SYBR green (#RT501M, Enzynomics, Daejeon, Korea) and primers (Supplementary Table S1) using the Stepone real-time PCR system (Applied Biosystems, Beverly, MA, USA).

To confirm the expression of the pluripotency markers, real-time quantitative-PCR was performed using TaqMan probes (SOX2 (#Hs00602736_s1, Thermo, Waltham, MA, USA), Nanog (#Hs02387400_g1,Thermo, Waltham, MA, USA) or Oct3/4 (#Hs04260367_Gh, Thermo, Waltham, MA, USA)) and pluripotency genes (SOX2, Nanog, Rex1, Oct3/4)

### 2.6. Alkaline Phosphatase (AP) Staining and Karyotype Analysis

According to the manufacturer’s instructions (Alkaline phosphatase stain kit (#AP100R-1, System Biosciences, Palo Alto, CA, USA), the stemness was validated by alkaline phosphatase (AP) staining. As for the karyotype analysis, GTG-binding analysis was performed in order to confirm the normal karyotype using each iPSC line (Gendix Inc., Seoul, Korea).

### 2.7. In Vivo Teratoma Formation

iPSC cultures at 1–2 × 10^6^ cells were prepared by mixing the cells in the media with Matrigel at a 1:1 ratio and injecting this into the shoulders or sides of NOD-scid gamma (NSG) mice using a BD ultra-fine II syringe (#328820, BD, Seoul, Korea). Teratomas were formed for 9–12 weeks after grafting and fixed in 4% paraformaldehyde overnight. According to standard procedures [18], paraffin-embedded sections were stained using hematoxylin and eosin (H&E staining) and the three germ layers were analyzed.

### 2.8. RNA Extraction and Sequencing 

To perform transcriptome sequencing (RNA-Seq) analysis, total RNA was extracted from the iPSC-derived CS neurons and control neurons without mice astrocytes. The quality and integrity of the extracted total RNA were assessed using BioAnalyzer and the standard Illumina sequencing system protocol (TruSeq RNA sample preparation kit v2) was used to make libraries for RNA-Seq. Approximately 300-bp-long fragments were isolated using gel electrophoresis; they were then amplified by PCR and sequenced on the Illumina HiSeq 4000 in the paired-end sequencing mode (2 × 101 bp reads).

### 2.9. RNA-Seq Data Analysis

The quality of the raw sequencing reads was assessed, and the sequencing reads were aligned to the human genome reference, hg19, using the HISAT2 alignment tool (version 2.1.0) [19]. Uniquely and properly mapped read pairs were used for further analysis. Gene annotation information was downloaded from Ensembl (release 92) biomart (http://www.ensembl.org/). Principal component analysis (PCA) was performed using one of the analysis modules in BioJupies [20]. The DESeq2 R package [21] was used to identify differentially expressed genes between iPSC-derived CS neurons and control neurons. Differentially expressed genes were defined as those with at least 2-fold changes between samples at a false discovery rate (FDR) of 5%. To classify the differentially expressed genes into protein functional classes, PANTHER (Protein ANalysis THrough Evolutionary Relationships) tools (http://www.pantherdb.org/) were used. Gene ontology (GO) term analysis and the Kyoto Encyclopedia of Genes and Genomes (KEGG) pathway enrichment analysis were performed using DAVID (Database for Annotation, Visualization, and Integrated Discovery) functional annotation tools [22]. 

### 2.10. Data Availability 

The transcriptome sequencing data (RNA-Seq) have been deposited at NCBI GenBank under BioProject ID PRJNA607582 (BioSample SAMN14140715–SAMN14140720). Please check data using the private reviewer link, https://dataview.ncbi.nlm.nih.gov/object/PRJNA607582?reviewer=sjrjfnt94bacqcqne39nah8pue.

### 2.11. Electrophysiology

Whole-cell patch clamp recording was performed [23] from induced neurons (iN) in a recording chamber that was perfused with a bath solution (pH 7.4) containing 140-mM NaCl, 5-mM KCl, 10-mM HEPES, 2-mM CaCl_2_, 2-mM MgCl_2_, and 10-mM glucose. The basic electrical properties, such as resting membrane potential, input resistance, and sodium and potassium currents, were recorded in the voltage or current-clamp mode. The intrinsic firing properties were recorded in the current-clamp mode at a membrane potential of −60 mV by injecting a current in a stepwise manner for 500 ms. 

### 2.12. Western Blot Analysis

We followed the protocol as described previously [24]. Briefly, proteins were extracted using a radioimmunoprecipitation assay (RIPA) buffer with a phosphatase inhibitor cocktail (#78426, Thermo, Waltham, MA, USA) and a protease inhibitor cocktail (#11836170001, Roche, Basel, Switzerland). The concentration of the proteins was determined using the bicinchoninic acid (BCA) protein assay reagent. The proteins were separated using sodium dodecyl sulfate-polyacrylamide gel electrophoresis (SDS-PAGE) and transferred onto polyvinylidene fluoride (PVDF) membranes (#IPVH00010, Millipore, Burlington, MA, USA). After blocking at room temperature for 1 h using 5% skim milk, the membrane was incubated overnight with primary antibodies at 4 °C and horseradish peroxidase (HRP)-conjugated secondary antibodies at room temperature for 1 h. Specific protein signals were visualized by the incubation of the enhanced chemiluminescence (ECL) solution (#WBKLS0100, Millipore, Burlington, MA, USA).

### 2.13. Generation of Neurospheres from CS iPSCs

Neurospheres from the CS iPSCs were generated as previously described [25], with minor modifications. Briefly, each iPSC was dissociated by treatment with accutase and the cells were plated at 10,000 cells into each well of an ultra-low attachment 96-well plate using a neural induction medium (DMEM-F12, 15% knockout serum replacement, 1% MEM-NEAA, 1% GlutaMAX, 100-μM beta-mercaptoethanol, 100-nM LDN-193189, 10-μM SB431542, and 2-μM XAV939) with 50-μM Y27632 and 5% FBS. On day 2, the medium was changed to the neural induction medium without 5% FBS and supplemented with 50-μM Y27632. On day 4, the medium was changed to the neural induction medium (without 5% FBS and 50-μM Y27632). Until day 10, the medium was changed daily, and the cells were cultured in a 37 °C incubator with 5% CO_2_. After day 10, the neurospheres were transferred into an ultra-low attachment 6-well plate containing the neural differentiation medium (1:1 mixture of DMEM-F12 media and neurobasal media, 0.2% N2 supplement, 1% B27 supplement without vitamin A, 1% GlutaMAX, 0.5% MEM-NEAA, 0.025% human insulin solution, 50-μM beta-mercaptoethanol, and 1% penicillin/streptomycin) on an orbital shaker with a speed of 80 rpm/min. The medium was changed every day until day 18. From day 18 onward, the neural differentiation medium was supplemented with vitamin A, 20-ng/mL BDNF, 200-μM cyclic adenosine monophosphate (cAMP), and 200-μM ascorbic acid and changed every 4 days [25].

## 3. Results

### 3.1. Clinical Features and Characterization of Mutations in the VPS13B Gene from a Cohen Syndrome Patient 

**Figure 1:**
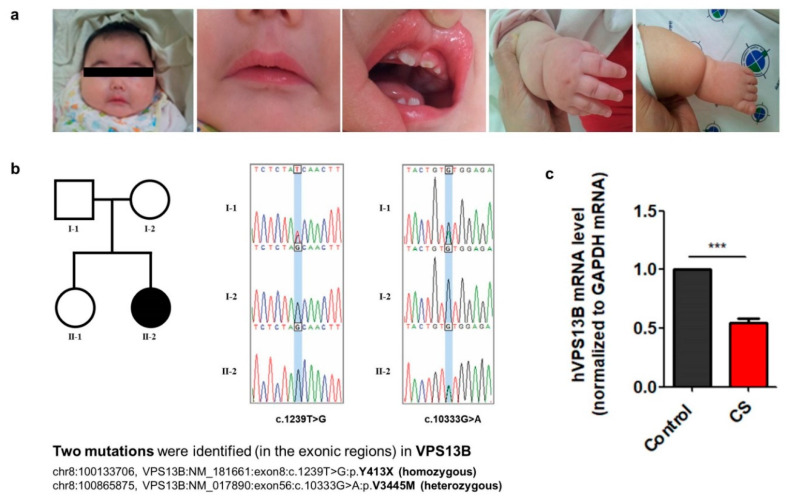
Clinical features and characterization of mutations in the *VPS13B* gene from a Cohen syndrome patient. (**a**) The 20-month-old female patient had the following features of Cohen syndrome at birth: microcephaly, frontal bossing, prominent nose, thick hair and eyebrows, micrognathia, cleft palate, glossoptosis, enamel hypoplasia of teeth, obesity, short limbs, and small hands and feet. (**b**) *VPS13B* gene mutations in a proband. Trio Sanger sequencing showed that a novel compound heterozygous mutation formed by homozygous variation c.1239T>G (p.Y413X) of biparental origin (I-1 and I-2) and heterozygous variation c.10333G>A (p.V3445M) inherited from the father (I-1) in the *VPS13B* gene were present in the proband. (**c**) Gene expression levels of *VPS13B* normalized with mRNA of glyceraldehyde-3-phosphate dehydrogenase (GAPDH). The relative level of *VPS13B* gene expression quantified by qPCR was significantly decreased in the proband as compared to control (Student t-test, *** *p* < 0.001).

The patient was born at 38 weeks’ gestation by spontaneous vaginal delivery, with a birth weight of 2.69 kg and a normal head circumference. The prenatal care and delivery were uneventful, but the patient was referred to the neonatal intensive care unit (NICU) because of poor feeding. The patient was hypotonic and showed the Pierre–Robin sequence (cleft palate, micrognathia, and glossoptosis). Feeding and breathing difficulties related to hypotonia lasted for about 2 weeks and then resolved. The hearing test showed sensory neural hearing loss in both ears. The tandem mass screening for metabolic disorders and chromosomal microarray was normal. During a regular visit at 6 months of age, global developmental delay, short stature (<3 percentile), a high body mass index (BMI) of 25 kg/m^2^, and microcephaly (<3 percentile) were noted (Figure 1). The patient had distinctive facial features including wave-shaped palpebral fissures, thick eyebrows, long eyelashes, short philtrum with an open-mouth appearance, and enamel hypoplasia in deciduous teeth. The extremities were short, particularly the legs, and the hands and feet were very small (Figure 1a). Whole-exome sequencing of the patient’s DNA revealed two mutations in *VPS13B*: c.1239T>G (p.Y413X) and c.10333G>A (p.V3445M). Trio Sanger sequencing of DNA from the patient, her parents, and her sister revealed that the c.1239T>G (p.Y413X) mutation was inherited from both parents and that the c.10333G>A (p.V3445M) mutation was inherited from the father (Figure 1b). Quantitative real-time PCR analysis showed that the mRNA level of *VPS13B* was slightly reduced in CS fibroblasts carrying the two mutations compared with control fibroblasts (Figure 1c).

### 3.2. Generation of CS-Derived iPSC and Rapid Neuronal Induction of CS iPSCs by Neurogenin2 (NGN2)

**Figure 2:**
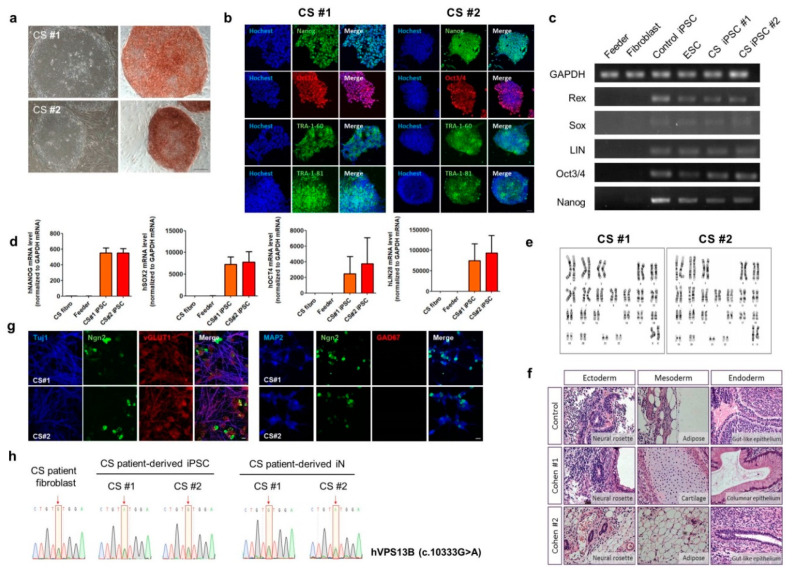
Generation of Cohen syndrome (CS)-derived induced pluripotent stem cells (CS #1, or CS#2 iPSCs) and neuronal differentiation from CS iPSCs. (**a**) Morphology of iPSC colonies and alkaline phosphatase staining in iPSCs. Scale bar, 200 μm. (**b**) Immunostaining of CS iPSCs with specific antibodies against intracellular pluripotent stem cell markers (Oct3/4, SSEA3, SSEA4, TRA1-60, and TRA1-81) in iPSCs. Scale bar, 50 μm. (**c**,**d**) Real-time PCR (RT-PCR) (semi- or quantitative RT-PCR) analysis of the expression of pluripotency markers (Oct3/4, Sox2, Rex1, and Nanog) in CS iPSCs. Bar graph presents mean ± SEM. (**e**) Normal karyotypes of CS iPSCs. (**f**) In vivo teratoma formation in nude mice. (**g**) Immunocytochemical analysis of CS induced neurons (iN) differentiated from CS iPSCs using a glutamatergic marker, VGLUT1, a GABAergic marker, GAD67, a neuronal marker, Tuj1, and MAP2. Scale bar, 5 μm. (**h**) Genomic sequence of the *VPS13B* gene in CS fibroblasts, CS iPSCs, and iPSC-derived neurons showing a c.10333G>A.

One of the major symptoms of CS is intellectual disability, which is characterized by significant impairment in intellectual ability or adaptive behavior [26]. To investigate the cellular pathogenesis of the intellectual disability of CS specifically in neurons, primary fibroblasts from the CS patient and a healthy control individual were reprogrammed into two different CS iPSC lines (#1 and #2) and the control iPSC line, respectively (Figure 2, Supplementary Figure S1). The pluripotency of these iPSC lines was validated by alkaline phosphate (AP) staining (Figure 2a), immunocytochemistry (Figure 2b), quantification of gene expression of pluripotent stem cell markers (Rex, Sox, LIN28, Oct3/4, or Nanog) using semi- (Figure 2c) or quantitative real-time (RT)-PCR (Figure 2d). Each iPSC line was also analyzed for karyotypic stability and in vivo differentiation (Figure 2e,f, Supplementary Figure S1a–c). In addition, the genetic mutations in the VPS13B gene, that were identified by the exome sequencing of the genomic DNA extracted from the blood samples of the CS patient, were confirmed in the CS fibroblasts or CS patient-derived iPSCs by Sanger sequencing (Figure 2h, Supplementary Figure S1e). 

Interestingly, the functional categorization of the genes associated with intellectual disability revealed the significant enrichment of the genes affecting glutamatergic synaptic structures and functions [27]. Therefore, for the functional characterization of the neurons derived from the CS patient, CS iPSCs or control iPSCs were induced into forebrain-like glutamatergic excitatory neurons using the NGN2-mediated rapid induction protocol [14] with slight modifications, as described in the Materials and Methods section (Figure 2g, Supplementary Figure S1d). We found that there were no significant differences in the numbers of dead cells between the control, CS #1, and CS #2 neurons 4 weeks after rapid neural induction by counting the 4′,6-diamidino-2-phenylindole (DAPI)-positive cells with nuclear condensation as the dead cells among the total number of DAPI-positive cells (% of dead cells: control, 5.6 ± 0.6; CS#1, 7.4 ± 1.7; CS#2, 6.5 ± 1.0%, One way AVOVA, Tukey post-hoc test, not significant between control, CS#1, and CS#2).

As shown in Figure 2g, Supplementary Figure S1d, and Supplementary Figure S2a–c, control or CS neurons 4 weeks after rapid neuronal induction were induced into Tuj1-, MAP2- or, VGLUT1-positive glutamatergic neurons but not GAD67-positive GABAergic neurons. When we counted the number of Tuj1-positive glutamatergic neurons among the total NGN2-positive neurons, there were no apparent differences in the rapid neuronal induction efficiency of the iPSCs between the control and CS neurons that had been co-cultured with mouse astrocytes (Supplementary Figure S2a,b). Moreover, when we counted the number of VGLUT1-positive neurons among the total Tuj1-positive neurons, there was no significant difference in the induction efficiency of the iPSCs into glutamatergic neurons between the control and CS (#1 or #2) neurons that had been co-cultured with mouse astrocytes (Supplementary Figure S2c). Moreover, there was no significant change in the basic electrophysiological properties, such as resting membrane potential, capacitance, input resistance, Na^+^, or K^+^ channel currents, membrane excitability and action potential properties of the CS-derived glutamatergic neurons as compared to the control neurons which had been co-cultured with mouse astrocytes (Supplementary Figure S3). We generated CS iPSCs which were rapidly induced into functional glutamatergic neurons and we found that there were no apparent differences in the rapid neuronal conversion efficiency of the iPSCs into functional glutamatergic neurons or the basic electrophysiological properties between the control and CS iPSC-derived glutamatergic neurons.

### 3.3. Transcriptomic Analysis in CS iPSC-Derived Glutamatergic Neurons

**Figure 3:**
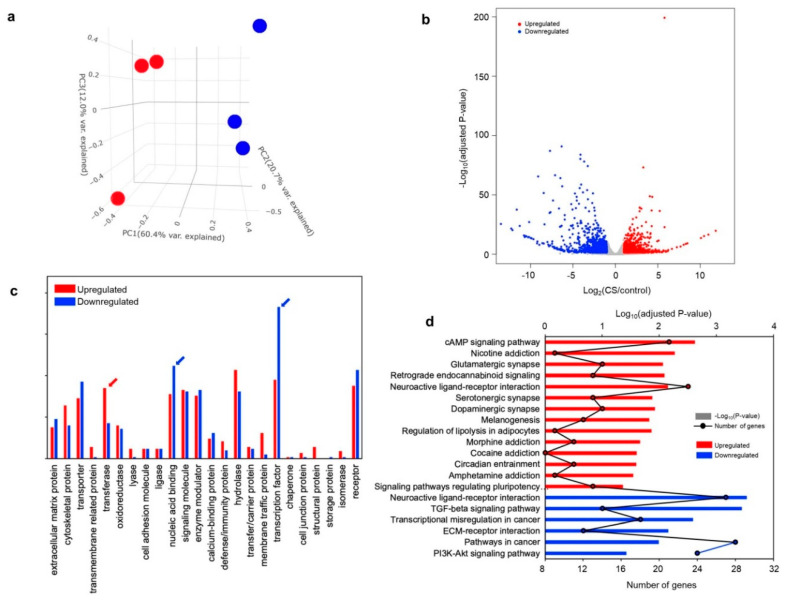
Differential gene expression analysis of iPSC-derived CS neurons. (**a**) Principal component analysis (PCA) of iPSC-derived CS neurons (red circles) and control neurons (blue circles) showing gene expression profiles for different samples (three biological replicates in each group). (**b**) A volcano plot shows differentially expressed genes. Red dots represent the significantly upregulated genes and blue dots represent the significantly downregulated genes in the iPSC-derived CS neurons. The X-axis represents the log_2_-transformed gene expression in iPSC-derived CS neurons divided by that in iPSC-derived control neurons. The Y-axis is the *p*-value (−log_10_) adjusted by the Benjamini–Hochberg correction. (**c**) Protein functional classification in differentially expressed genes was performed using the PANTHER (Protein ANalysis THrough Evolutionary Relationships) tool. Red (upregulated) and blue (downregulated) arrows indicate protein functional classes that show significantly different composition (more than 3% composition difference) between iPSC-derived CS neurons and control neurons. (**d**) Kyoto Encyclopedia of Genes and Genomes (KEGG) pathway enrichment in genes upregulated (red) and downregulated (blue) in iPSC-derived CS neurons. The adjusted *p*-values of significantly enriched terms or pathways and the number of the genes in the KEGG pathways are shown as bar plots (−log_10_ adjusted *p*-value) and line graphs (number of genes in the specific enriched pathways).

To gain insight into the cellular pathogenesis of intellectual disability in CS patient-derived neurons, we performed RNA-Seq-based transcriptomic analysis and subsequent quantitative RT-PCR (qRT-PCR) analysis on the iPSC-derived control and CS neurons without mice astrocytes. Three biological replicates were collected from the iPSC-derived CS neurons and control neurons. A total of 238 million pairs of reads (2 × 101 bp) were obtained using Illumina HiSeq 4000. More than 82% of the read pairs could be mapped on the human genome (hg19) uniquely (Supplementary Table S2). A total of 32,888 genes (Ensembl genes release 92) had at least one read mapped on the exonic regions. Principal component analyses (PCA) revealed that 93.1% of the variations in the RNA sequencing data could be explained by the first three principal components (PCs), and, as shown in Figure 3a, the first PC isolates in the three biological replicates according to each group (control and CS). Differentially expressed genes between the control and CS neurons were identified using DESeq2 [21]. The analysis revealed a total of 2039 differentially expressed genes (DEGs) (cutoffs: |Fold change| ≥ 2, FDR 5%), out of which 1007 were upregulated and 1032 were downregulated (Figure 3b, Supplementary Table S3). 

To investigate the functional categories of DEGs, we classified the DEGs into PANTHER (Protein ANalysis THrough Evolutionary Relationships) protein class categories (Figure 3c). The DEGs identified in each comparison were divided into a total of 23 different protein classes. The fraction of “transferase” class DEGs in the upregulated genes was larger in the CS group than in the control group. The fractions of “nucleic acid binding” and “transcription factor” classifications showed the opposite trend. Furthermore, functional annotation analyses using the Gene Ontology (GO) database and KEGG pathway analyses showed that synapses and signaling pathways, such as “postsynaptic membrane”, “glutamatergic synapse”, and “cAMP signaling pathway”, were enriched in the upregulated genes. Conversely, processes associated with transcriptional regulation and development, such as “positive regulation of cell proliferation”, “TGF-beta signaling pathway”, and “nervous system development”, were enriched in the downregulated genes, suggesting the dysregulation of cell proliferation or differentiation (Figure 3d, Table 1, and Supplementary Table S4). Recently, Zhou et al. [28] developed a method, metascape, to integrate and cluster the functional terms for the multiple and comprehensive enrichment test results. Moreover, this method can perform comparative enrichment tests for the multiple DEGs, for both upregulated and downregulated genes. Therefore, we decided to use these tools to perform comprehensive enrichment tests and compare the upregulated and downregulated genes. Surprisingly, both upregulated and downregulated genes were enriched in representative “Neuroactive ligand-receptor interactions” and “trans-synaptic signaling” (Supplementary Figure S4). Furthermore, the enrichment *p*-values for the terms in the downregulated genes were more significant than the ones in the upregulated genes (darker color in the heatmap in the Supplementary Figure S4) and the various development terms were enriched only in the downregulated genes, as expected.

### 3.4. Alteration of Genes Involved in Synapse Function in CS iPSC-Derived Glutamatergic Neurons

Based on our functional annotation analyses for the KEGG pathways associated with upregulated or downregulated genes, we first investigated the target genes responsible for synapses in CS glutamatergic neurons by examining the differentially expressed genes, focusing on the glutamatergic synapse signaling pathway. Most genes associated with glutamatergic synapses, such as *SLC17A8*, *GRIN2B*, *GRIA1*, *GRM8*, *ADCY8*, *ADCY5*, *GRIK4*, *PRKACB*, *GRIN3A*, *PLA2G4C*, *PLCB2*, *SHANK2*, and *SV2B*, were altered in the CS-derived glutamatergic neurons (Table 1). In addition, genes related to the cAMP signaling pathway, such as *PTGER3*, *ADCY8*, *ADCY5*, *PDE3A*, *GRIN3A*, *TNNI3*, *VAV1*, *GRIN2B*, *GRIA1*, *PIK3R5*, *PRKACB*, *ARAP3*, *CAMK2A*, *AKT3*, and *HTR1E*, were increased in CS neurons as compared to control neurons (Table 1). However, the genes that function in cancer, the TGF-β signaling pathway, or neuroactive ligand receptor interaction were downregulated (Table 1).

**Figure 4:**
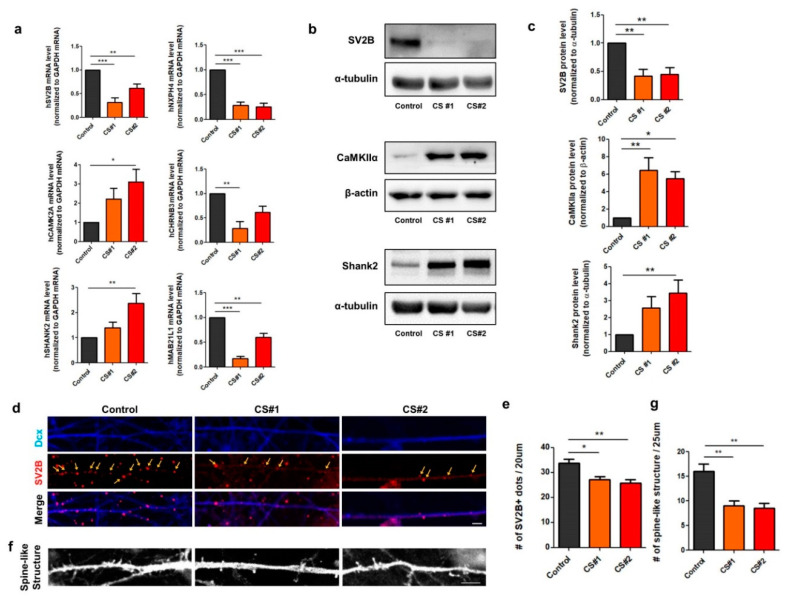
Alteration of gene expression related to synaptic function in CS iPSC-derived glutamatergic neurons. (**a**) Differential gene expression analysis of six representative genes (*NXPH4*, *SV2B*, *MAB21L1*, *CHRNB3*, *SHANK2*, and *CAMK2A*) associated with synaptic function using quantitative RT-PCR (qRT-PCR). Relative gene expression was normalized to mRNA of GAPDH. Bar graph presents mean ± SEM. (One-way AVOVA, Bonferroni post hoc test, * *p* < 0.05, ** *p* < 0.01, *** *p* < 0.001). (**b**) Western blots of SV2B, CaMKIIα, and Shank2 in control- and CS-derived neurons at 3-4 weeks after neuronal differentiation. Relative protein levels were normalized to α-tubulin. (**c**) Quantitative data from Western blot analysis. Bar graph represents mean ± SEM (One-way AVOVA, Bonferroni post hoc test, * *p* < 0.05, ** *p* < 0.01). (**d**) Representative images of SV2B-positive vesicles of neurons by immunostaining. Scale bar, 5 μm. (**e**) Quantification of SV2B-positive vesicles on doublecortin (DCX)-positive neurites per 20 μm. Bar graph represents mean ± SEM (One-way AVOVA, Bonferroni post hoc test, * *p* < 0.05, ** *p* < 0.01). (**f**) Representative images of green fluorescent protein (GFP)-positive spine structures of neurons. Scale bar, 5 μm. (**g**) Quantification of GFP-positive spine-like structures on neurites per 25 μm. Bar graph represents mean ± SEM (One-way AVOVA, Bonferroni post hoc test, ** *p* < 0.01).

To validate the alteration of gene expression that we observed using RNA-Seq analysis, we performed qRT-PCR analysis for the genes showing higher fold-changes in expression in CS-derived neurons compared to control neurons without mice astrocytes. We found a decreased expression of NXPH4, SV2B, MAB21L1, and CHRNB3 in the CS iPSC-derived glutamatergic neurons as compared with the control neurons, which was consistent with the results of the RNA-Seq analyses. Interestingly, the mRNA levels of SHANK2 and CAMK2A were significantly upregulated in the CS neurons as compared with the control neurons (Figure 4a), and in turn the protein levels of Shank2 and CaMKII were also significantly increased in the CS neurons as compared with the control neurons without mice astrocytes (Figure 4b). Furthermore, the number of SV2B-positive puncta or GFP-positive spine-like structures was significantly reduced in the neurites of the CS iPSC-derived neurons as compared with the control neurons on the mice astrocytes, thus leading to synaptic dysregulation in the CS iPSC-derived glutamatergic neurons (Figure 4d–g). Taken as a whole, our gene expression profiling suggests that alterations in the expression of genes associated with synapse function, such as SV2B, CAMK2A, or SHANK2, in CS glutamatergic neurons may contribute to cellular pathogenesis in CS patients.

### 3.5. Small Size of Neurospheres Differentiated from CS iPSCs 

**Figure 5:**
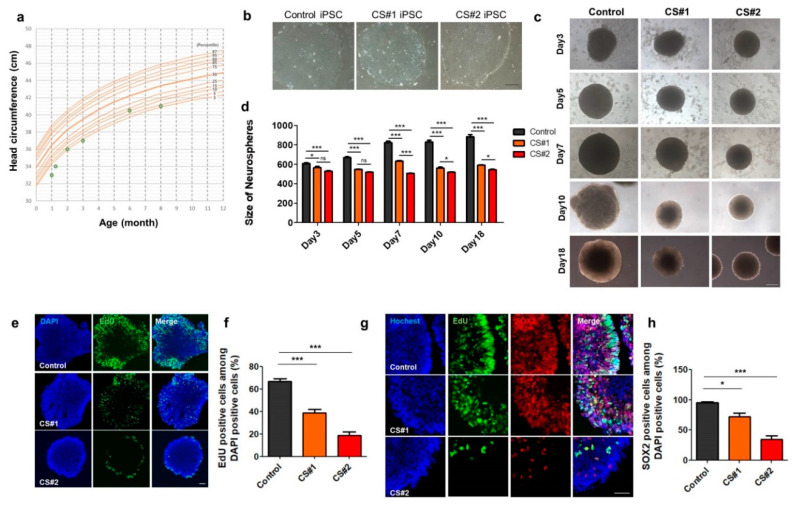
Small size of neurospheres differentiated from CS patient-derived iPSCs. (**a**) Growth curve of the CS patient. (**b**) Morphology of control or CS iPSC colonies. Scale bar, 200 μm. (**c**) Representative images of neurospheres (control or CS) from day 3 to day 18, scale bar, 200 μm. (**d**) Bar graph represents the size of neurospheres from day 3 to day 18. Bar graph represents mean ± SEM (One-way AVOVA, Tukey’s multiple comparison test, * *p* < 0.05, *** *p* < 0.001), ns, no significance. (**e**) EdU staining (proliferation marker, EdU) of control and CS neurospheres (day 7 after neural induction). (**f**) Quantification of cell proliferation of EdU-positive cells among DAPI-positive cells; bar graph represents mean ± SEM (One-way AVOVA, Bonferroni post hoc test, *** *p* < 0.001) (**g**) EdU staining and immunostaining of control or CS neurospheres (day 20 after neural induction) with SOX2 with anti-SOX2 antibody. Scale bar, 50 μm. (**h**) Quantification of SOX2-positive cells among total DAPI-positive cells; bar graph represents mean ± SEM (One-way AVOVA, Tukey’s multiple comparison test, * *p* < 0.05, ** *p* < 0.01).

Postnatal microcephaly is a well-defined clinical feature of CS patients [1,2]. The head circumference of our CS patient was 33 cm (<3 percentile) at one month after birth, while the mean head circumference at this age is normally 36.5 cm (50th percentile). Up to 8 months after the birth, the patient had a smaller-than-normal head circumference at almost under three percentiles of normal growth (Figure 5a). Interestingly, in our transcriptomic analysis of the CS iPSC-derived neurons, the processes associated with transcriptional regulations and development, such as the “positive regulation of cell proliferation”, “TGF-beta signaling pathway”, and “nervous system development”, were enriched in the downregulated genes (Figure 3d). Therefore, to investigate early cell proliferation, we differentiated CS iPSCs into 3-D neurospheres, as described previously [25]. 

Interestingly, as shown in Figure 5b, the size of the CS neurospheres was smaller than that of the control neurospheres at five days after neural induction (Figure 5c–e). More remarkably, the size of the CS neurospheres was smaller than that of the control neurospheres at 18 days after their differentiation (Figure 5d). To examine whether the proliferation property of the NPCs is altered, we further performed a cell proliferation assay using EdU staining in control or CS iPSC-derived neurospheres. Indeed, the number of EdU-positive NPCs was significantly less in the CS neurospheres as compared to the control neurospheres, thus indicating that the cell proliferation of the NPCs was significantly reduced at 7 days after neural induction in the CS neurospheres (Figure 5e,f). Furthermore, the Sox2-positive neural progenitor cells were significantly reduced in the CS neurospheres as compared to the control neurospheres (Figure 5g,h). 

Therefore, our small 3-D neurospheres for CS may reflect the cellular pathogenesis of the CS patient. We suggest a novel cellular pathogenesis pathway which shows the reduced proliferation of Sox2-positive NPCs during the early neural development of the CS patient.

## 4. Discussion

Cohen syndrome (CS) is an uncommon autosomal recessive developmental disorder that has been attributed to mutations in the *VPS13B* gene in patients of diverse genetic backgrounds [2]. Although most CS patients have several common features, like intellectual disability, postnatal microcephaly, or short stature [29], regardless of different mutations in the *VPS13B* gene and other factors, the clinical heterogeneity of CS is evident when comparing CS patients of different ethnic backgrounds [30]. Due to these reasons, to date, there have been few studies on the pathogenesis of CS using transgenic or knock-in mice models expressing different genetic mutations. Moreover, there is no human disease model representing CS patients with or without different mutations in the *VPS13B* gene, and its cellular pathogenic mechanism in human patient-derived neuronal disease models is largely unknown. In this study, we generated a personalized, patient-specific iPSC disease model from CS patients with *VPS13B* mutations in order to investigate the cellular pathogenic mechanism of CS.

Genetic analysis from exome sequencing showed that our CS patient had both a homozygous nonsense mutation (exon8:c.1239T>G:p.Y413X) and a heterozygous missense mutation (exon56:c.10333G>A:p.V3445M) in the exonic region of the *VPS13B* gene. Among these, c.10333G>A (p.V3445M) was inherited only from the father (Figure 1b, I-1). Although the combined or relative contribution of both mutations to the expression of the *VPS13B* gene is not clear in this CS patient, we found that the mRNA levels of *VPS13B* were slightly, but significantly, reduced in the CS fibroblasts and iPSCs as compared to the control (Figure 1c). This increases the possibility that the reduced expression of VPS13B partially contributes to the clinical features. It has been reported that patients with null mutations in *VPS13B* show the typical features of autism spectrum disorder (ASD), whereas milder forms of CS resulting from missense mutations are associated with microcephaly, mild dysmorphic features, and joint laxity, suggesting that the expression level of VPS13B affects the severity of clinical features in CS patients [30]. However, we cannot rule out the possibility that other genetic mutations contributed to the pathogenic features of this CS patient. In addition, in our study, we characterized only one CS patient-derived iPSC due to its rarity. To overcome this limitation of the current study, future work will aim to increase the sample size by generating patient-specific mutations in an isogenic line, using CRISPR/Cas9 technology to clarify the effect of each mutation in *VPS13B* on the cellular pathogenesis of CS. 

One of the major advantages of modeling a human disease using patient-derived iPSCs is that it allows the study of the pathophysiology in personalized, patient-derived specific cell-types, such as post-mitotic neurons, which is not otherwise possible without invasive dissection. Among the several clinical features of CS, we were interested in intellectual disability and microcephaly, which can be studied in forebrain-like glutamatergic neurons by using the rapid neuronal induction protocol and 3-D neurospheres differentiated from CS patient-derived iPSCs, respectively. In other previous studies, the aberrant glutamatergic synapse function in the etiology of intellectual disability, ASD, schizophrenia, and other neurodevelopmental disorders has been extensively reported [26]. Changes in the expression of many synaptic proteins have also been associated with disorders of the nervous system. Indeed, our functional annotation analyses showed that genes related to glutamatergic synapses and signaling were dysregulated (Supplementary Figure S4). However, we do not know whether this dysregulation of gene expression correlates with the intellectual disability of CS. We cannot exclude the possibility that the up- and downregulation of glutamatergic synapse genes in CS neurons might be due to the variability in the differentiation and maturation of the iPSC-derived neurons. Therefore, the best way to address this issue, in a future study, would be to generate isogenic iPSC lines or more iPSC clones and differentiate them into neurons for further analysis of synaptic dysregulation in CS neurons. 

Among synapse-related genes, the expression of *SHANK2*, *CAMK2A*, or *SV2B* was significantly altered at both the RNA and protein levels in the CS iPSC-derived glutamatergic neurons, suggesting synaptic dysregulation. SHANK/ProSAP proteins are essential for synaptic formation, development, and function [31]. Intriguingly, mutations in *SHANK2* are associated with ASD, neurodevelopmental disorders, and neuropsychiatric disorders, such as intellectual disability and schizophrenia [32]. In addition, calcium/calmodulin-dependent protein kinase II (CAMKII) is one of the first proteins that was shown to be essential for normal learning and synaptic plasticity in mice [33]. Recently, it has been reported that de novo mutations in the protein kinase genes *CAMK2A* and *CAMK2B* cause intellectual disability and neurodevelopmental disorders [34,35]. Another synapse-associated protein, synaptic vesicle glycoprotein 2B (SV2B), is localized to synaptic vesicles and functions in the regulation of vesicle trafficking, exocytosis, and calcium homeostasis in the presynaptic terminal, leading to the control of synaptic functionality [36,37], and it has been implicated in human epilepsy [37]. In our data, the CS neurons showed a reduced expression of SV2B, leading to fewer SV2B-positive puncta. However, further work is needed to determine whether the loss of SV2B also contributes to synaptopathy in the cellular pathogenesis of CS. Moreover, in this study, we also found that the number of spine-like structures was reduced in the CS neurons as compared to control neurons, raising the possibility of synaptic dysregulation in the CS pathogenesis. It is possible that the different regulations in upregulated and downregulated genes, functionally categorized as “neuroactive ligand-receptor interactions” and “trans-synaptic signaling” terms, as described in the Results, could affect the reduced spine-like structure phenotypes in the CS-derived neurons. To address this possibility, we need to further analyze other synapse-related genes and genes from various neuronal subtypes in a future study. 

Based on our study, we provide a CS glutamatergic neuronal model which represents the alteration of synapse-associated genes and propose that the dysregulation of major synapse-associated genes contributes to intellectual disability and ASD-like features in our CS patient. However, further investigation is required to discern whether this differential gene regulation in CS patient-derived neuronal cultures is related to the reduced cell proliferation that was observed in CS neurospheres.

The NGN2-mediated rapid neuronal induction protocol has several advantages for the production of a homogenous population of induced neurons and reduces the time required for neuronal induction or maturation for synaptic formation. However, the neurons rapidly generated by NGN2 expression could not reflect the whole process of neural differentiation or neuronal maturation or other subtype-specific neurons, including GABAergic, dopaminergic, or striatal neurons. Indeed, NGN2-positive neurons are mostly excitatory glutamatergic neurons but not GABAergic neurons in our study and in the paper published by Zhang et al. [14]. In addition, the rapid induction protocol could not reflect the early neural differentiation process due to its direct conversion of iPSCs into functional neurons. Moreover, in vivo brain neural differentiation occurs in 3-D, but not 2-D, structures. Therefore, considering the high resemblance of 3-D neurospheres, or organoid models, to the early stages of neurogenesis, they may be the best models for developmental disorders presenting at the embryonic or fetal stages [38,39]. A few examples are provided by studies that report the use of iPSCs derived from microcephalic patients with genetic mutations or those infected by Zika virus [40,41,42]. 

In our study, there is a conflicting issue of neural differentiation between neurospheres and the NGN2-based rapid iN protocol. Our CS neurospheres were smaller than the control neurospheres at five days after neural induction; however, there was a similar rapid neuronal conversion efficiency of the CS iPSCs into glutamatergic neurons when compared with that of the control iPSCs. This discrepancy may be derived from the differences between the two protocols. The rapid induction protocol via NGN2 expression may not reflect the entire process of early neural differentiation, owing to its direct conversion of iPSCs into neurons [43,44,45]. This discrepancy could be addressed by a conventional neural induction protocol that may reflect the entire process of early neural differentiation, including glutamatergic or GABAergic neurons.

In addition, we hypothesize that there may be a line variation difference between CS1 and CS2 iPSC-derived cells at the cellular phenotype level, although we could not determine the cause of the difference in the sizes of CS1 and CS2 neurospheres. However, regardless of the differences in size between the CS1 and CS2 neurospheres, the reduced proliferation of Sox2-positive NPCs may contribute to the small size of the CS patient-derived neurospheres (both CS1 and CS2 neurospheres), at least in our in vitro culture model. In neurogenesis, SOX2 is expressed throughout the developing cells in the neural tube, as well as in proliferating central nervous system progenitors [46]. It has been reported that SOX2 transcriptionally regulates PQBP1, an intellectual disability–microcephaly causative gene, in neural stem progenitor cells (NSPCs) [47]. PQBP1 is a nuclear-cytoplasmic shuttling protein that is engaged in RNA metabolism and transcription. Its expression patterns in NSPCs are related to the symptoms of intellectual disability and microcephaly in *PQBP1* gene-mutated patients and its exogenous expression rescued microcephalic phenotypes [48]. Thus, it is possible that reduced SOX2 levels result in the dysregulated expression of PQBP1, which may be associated with the smaller size of neurospheres. However, the more detailed cellular pathogenic mechanisms regarding how the proliferation of SOX2-positive neural progenitor cells are reduced in the CS neurospheres must be investigated further. 

We aimed to determine the relationship between the reduced expression of VPS13B in CS patient-derived cells and the dysregulation of NPC proliferation or alteration of synapse-related genes/synaptic structures. VPS13 has been implicated in various processes, including vesicle fusion, autophagy, and actin regulation in yeast and other organisms [3,49,50,51,52,53]. Mammalian VPS13B regulates endosome–lysosome trafficking or glycosylation in the Golgi complex [54]. Several receptors and membrane proteins play important roles in NPC proliferation or synapse formation and maturation which require proper trafficking. In addition, several membrane proteins, such as SV2B, are glycosylated, which is essential for proper function. Thus, reduced VPS13B protein trafficking may affect cellular localization or the degradation of several membrane proteins/receptors which are essential for the proliferation of NPCs or synaptic function.

Our study provides a human CS patient-derived neuronal model and 3-D neurosphere model system in which there is an alteration of synapse-associated gene expression and reduced proliferation of NPCs. These models can help us to develop new therapeutic drugs and facilitate therapeutic interventions for CS patients. 

## Figures and Tables

**Table 1:** Enriched KEGG pathways.

KEGG Pathway	Number of Genes	Adjusted *p*-Value	Genes
**Upregulated**			
cAMP signaling pathway	21	0.00236638	*FXYD2, PTGER3, ADCY8, ADCY5, PDE3A, GRIN3A, TNNI3, VIPR2, VAV1, BDNF, GRIN2B, PPP1R1B, GRIA1, HTR6, PIK3R5, PRKACB, ARAP3, HTR1D, CAMK2A, AKT3, HTR1E*
Nicotine addiction	9	0.00529695	*SLC17A8, GABRG2, GABRE, GRIN2B, GABRA4, GRIA1, GABRB2, GRIN3A, GABRQ*
Glutamatergic synapse	14	0.00864085	*ADCY8, ADCY5, GRIK4, GRIN3A, SHANK2, SLC17A8, GRIN2B, GRM8, GRIA1, SLC1A7, PRKACB, PLA2G4C, PLCB2, PLA2G4D*
Retrograde endocannabinoid signaling	13	0.00803098	*SLC17A8, GABRG2, GABRE, KCNJ9, GABRA4, GRIA1, ADCY8, GABRB2, ADCY5, FAAH, PRKACB, PLCB2, GABRQ*
Neuroactive ligand-receptor interaction	23	0.00703938	*GABRG2, GABRE, PTGER1, PTGER3, THRB, GABRA4, DRD3, GABRB2, CYSLTR2, GRIK4, TSHB, GRIN3A, VIPR2, CRHR2, GRIN2B, GRM8, GRIA1, HTR6, MAS1, CALCRL, HTR1D, GABRQ, HTR1E*
Serotonergic synapse	13	0.01312029	*KCND2, KCNJ9, GABRB2, ADCY5, HTR6, PTGS1, MAOB, PRKACB, HTR1D, PLA2G4C, PLCB2, HTR1E, PLA2G4D*
Dopaminergic synapse	14	0.01189212	*CALY, DRD3, PPP2R5A, ADCY5, TH, MAOB, GRIN2B, KCNJ9, GRIA1, PPP1R1B, PRKACB, CAMK2A, PLCB2, AKT3*
Melanogenesis	12	0.01494844	*WNT5A, WNT4, ADCY8, ADCY5, FZD1, PRKACB, WNT7A, CAMK2A, PLCB2, TCF7L2, WNT8A, WNT2B*
Regulation of lipolysis in adipocytes	9	0.01360123	*IRS4, PTGER3, ADCY8, ADCY5, PTGS1, TSHB, PIK3R5, PRKACB, AKT3*
Morphine addiction	11	0.02151968	*GABRG2, GABRE, PDE7B, KCNJ9, GABRA4, ADCY8, GABRB2, ADCY5, PDE3A, PRKACB, GABRQ*
Cocaine addiction	8	0.02474648	*BDNF, GRIN2B, PPP1R1B, ADCY5, MAOB, TH, PRKACB, GRIN3A*
Circadian entrainment	11	0.02502941	*RPS6KA5, NOS1, GRIN2B, KCNJ9, GRIA1, ADCY8, ADCY5, GUCY1A2, PRKACB, CAMK2A, PLCB2*
Amphetamine addiction	9	0.02830907	*GRIN2B, GRIA1, PPP1R1B, ADCY5, MAOB, TH, PRKACB, GRIN3A, CAMK2A*
Signaling pathways regulating pluripotency of stem cells	13	0.04332556	*WNT5A, FZD1, POU5F1B, WNT2B, PCGF5, WNT4, POU5F1, PIK3R5, JAK3, FGF2, WNT7A, WNT8A, AKT3*
			
**Downregulated**			
Neuroactive ligand-receptor interaction	27	2.93E-04	*CGA, OPRK1, GLRA2, BDKRB2, S1PR3, APLNR, CHRNA9, GALR1, CHRNA5, CHRNA6, CHRNA1, CHRNA3, GABRG3, GABRA1, PTH2R, GRM4, GABRR1, CHRM4, CHRM3, PRLR, GRM6, MC4R, CHRNB4, ADRA1A, GPR50, CHRNB3, CHRNG*
Transforming growth factor (TGF)-beta signaling pathway	14	3.57E-04	*SMAD9, SMAD7, TGFBR2, TGFB3, TGFB1, ID2, INHBE, ID3, BAMBI, MYC, CHRD, BMP5, PITX2, ACVR1*
Transcriptional misregulation in cancer	18	0.00256055	*LMO2, TGFBR2, RXRG, SIX4, ZBTB16, MEIS1, FEV, JUP, CDKN1A, ID2, NTRK1, PAX8, SIX1, ETV1, NGFR, TLX3, MYC, TLX1*
Extracellular Matrix (ECM)-receptor interaction	12	0.00695211	*ITGA9, COL6A6, COL27A1, TNC, ITGA8, COL3A1, ITGB6, SV2B, COL2A1, LAMB1, COL5A2, CHAD*
Pathways in cancer	28	0.01023292	*FGF18, ADCY2, PGF, TGFB3, KITLG, FGF10, FGF13, ZBTB16, BDKRB2, MMP2, TGFB1, TCF7L1, FLT3LG, CXCR4, PAX8, TGFA, LAMB1, MYC, EGFR, FZD8, EPAS1, TGFBR2, RXRG, MECOM, JUP, CDKN1A, FZD10, NTRK1*
PI3K-Akt signaling pathway	24	0.03734476	*EGFR, FGF18, PGF, TNC, COL3A1, KITLG, FGF10, COL2A1, FGF13, COL5A2, CHAD, ITGA9, CDKN1A, COL6A6, PRLR, ITGA8, COL27A1, IL4R, ITGB6, PDGFC, NGFR, LAMB1, MYC, IL3RA*

## Supplementary Materials

The following are available online at https://www.mdpi.com/2077-0383/9/6/1886/s1, Figure S1: Generation of control iPSC and neuronal differentiation from the control iPSCs. Figure S2: Neuronal differentiation from control and CS iPSCs. Figure S3: Basal electrophysiological properties in CS iPSC-derived glutamatergic neurons. Figure S4: A heatmap visualization of metascape results based on upregulated and downregulated gene lists. Table S1: Information of quantitative real-time PCR primers. Table S2: RNA-Seq read mapping summary. Table S3: A list of differentially expressed genes between iPSC-derived CS neurons and control neurons. Table S4: A list of GO terms enriched in upregulated and downregulated genes.
