# Supplementary material for: Cohen Syndrome Patient iPSC-Derived Neurospheres and Forebrain-Like Glutamatergic Neurons Reveal Reduced Proliferation of Neural Progenitor Cells and Altered Expression of Synapse Genes
Source: J Clin Med. 2020 Jun 16;9(6):1886. doi: 10.3390/jcm9061886 (PMC7356975; doi:10.3390/jcm9061886)
Supplement: Supplementary file 1 [file jcm-09-01886-s001.zip › Figure S3.pdf]

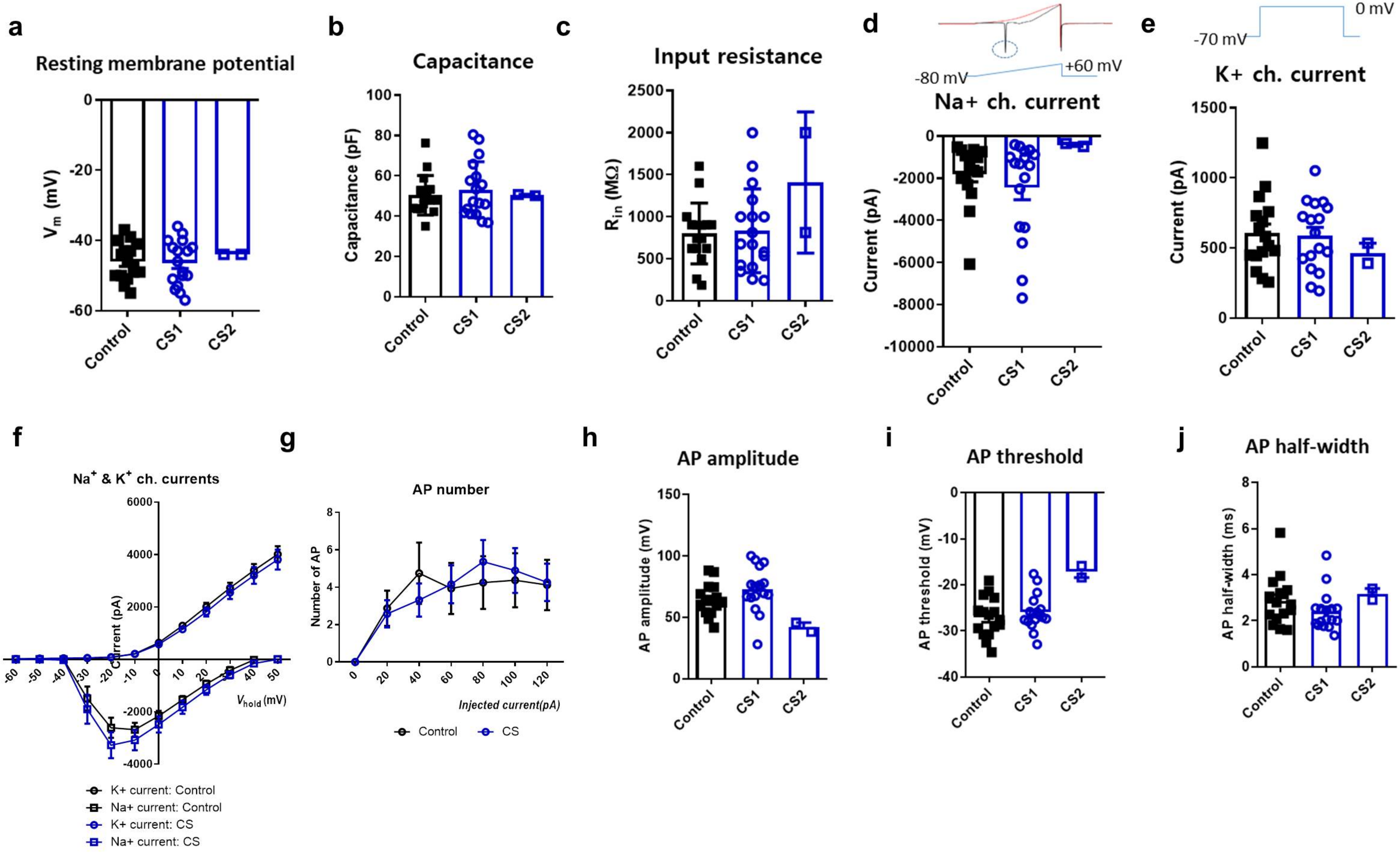

a
Resting membrane potential
Vm (mV)
Control
CS1
CS2
b
Capacitance
Capacitance (pF)
c
Input resistance
Rin (MΩ)
d
-80 mV
+60 mV
Na+ ch. current
Current (pA)
e
-70 mV
0 mV
K+ ch. current
Current (pA)
f
Na+ & K+ ch. currents
Current (pA)
Vhold (mV)
K+ current: Control
Na+ current: Control
K+ current: CS
Na+ current: CS
g
AP number
Number of AP
Injected current(pA)
Control
CS
h
AP amplitude
AP amplitude (mV)
i
AP threshold
AP threshold (mV)
j
AP half-width
AP half-width (ms)
