# Supplementary material for: Cohen Syndrome Patient iPSC-Derived Neurospheres and Forebrain-Like Glutamatergic Neurons Reveal Reduced Proliferation of Neural Progenitor Cells and Altered Expression of Synapse Genes
Source: J Clin Med. 2020 Jun 16;9(6):1886. doi: 10.3390/jcm9061886 (PMC7356975; doi:10.3390/jcm9061886)
Supplement: Supplementary file 1 [file jcm-09-01886-s001.zip › Table S1.docx]

**Supplementary Table 1**. **Real-time PCR primers**

| **Primer** | **Sequence** |
| --- | --- |
| hSV2B-S | 5’-TTT GGC AAC AGT GAG TCT GC-3’ |
| hSV2B-A | 5’-AGA GAA GCA GCA GCC AGA AG-3’ |
| hShank2-S | 5’-GCT TTG GAT TCG TGC TTC G-3’ |
| hShank2-A | 5’-GTA CTG TAG GGC TGG GAA AG-3’ |
| hCaMKIIa-S | 5’-GAG CTG CCA GTT CCC TAC AG-3’ |
| hCaMKIIa-A | 5’-VTAG CCA GAC GTT TGC TCC TT-3’ |
| hNXPH4-S | 5’-CCT GTC CCC TTT TCC TCC AA-3’ |
| hNXPH4-A | 5’-CCC TTT CCC GTC TGT TTT GG-3’ |
| hCHRNB3-S | 5’-TGC CCT CCT CAG ACA TTT GT-3’ |
| hCHRNB3-A | 5’-TCC AGC GTA ACT TGT GGT CT-3’ |
| hMAB21L1-S | 5’-GGA GGC AGA GAA CAG ACT GC-3’ |
| hMAB21L1-A | 5’-GCA GGC AGG AGA TAA GTT GC-3’ |
| hSOX2-S | 5’-GGG AAA TGG GAG GGG TGC AAA AGA GG-3’ |
| hSOX2-A | 5’-TTG CGT GAG TGT GGA TGG GAT TGG TG-3’ |
| hNanog-S | 5’-AAG ACA AGG TCC CGG TCA AG-3’ |
| hNanog-A | 5’-CAG GCA TCC CTG GTG GTA G-3’ |
| hRex1-S | 5’-AAG GCA AGT CAA GCC AAG ACC-3’ |
| hRex1-A | 5’-TTC CAA AGA ACA TTC AAG GGA GC-3’ |
| hOct3/4-S | 5’-CCC CAG GGC CCC ATT TTG GTA CC-3’ |
| hOct3/4-A | 5’-ACC TCA GTT TGA ATG CAT GGG AGA GC-3’ |
| hLIN28-S | 5’-GCG AAC CCA AGA CCC AGG CCT GCT CCV |
| hLIN28-A | 5’-CAG GGG GTC TGC TCG CAC CGT GAT G-3’ |
| hGAPDH-S | 5’-GGA GCG AGA TCC CTC CAA AAT-3’ |
| hGAPDH-A | 5’-GGC TGT TGT CAT ACT TCT CAT GG-3’ |
| hVPS13B-S | 5’-GGT TGG CAG TCA GGA CAT TT-3’ |
| hVPS13B-A | 5’-ATC CAT GCT GCT GAT GAG TG-3’ |
