# Supplementary material for: Cohen Syndrome Patient iPSC-Derived Neurospheres and Forebrain-Like Glutamatergic Neurons Reveal Reduced Proliferation of Neural Progenitor Cells and Altered Expression of Synapse Genes
Source: J Clin Med. 2020 Jun 16;9(6):1886. doi: 10.3390/jcm9061886 (PMC7356975; doi:10.3390/jcm9061886)
Supplement: Supplementary file 1 [file jcm-09-01886-s001.zip › Table S2.docx]

**Supplementary Table 2. RNA-Seq read mapping summary**

| **Samples** | | **Total raw sequencing read pairs** | **Uniquely mapped read pairs** | **Unmapped read pairs** | **% of uniquely mapped read pairs** |
| --- | --- | --- | --- | --- | --- |
| Control neurons | Replicate 1 | 37,853,649 | 31,712,219 | 4,474,171 | 83.78 |
|  | Replicate 2 | 35,630,324 | 29,308,125 | 4,474,202 | 82.26 |
|  | Replicate 3 | 41,037,986 | 34,893,525 | 4,225,267 | 85.03 |
| CS neurons | Replicate 1 | 45,485,637 | 38,354,243 | 5,127,303 | 84.32 |
|  | Replicate 2 | 37,932,889 | 32,90,1165 | 3,285,581 | 86.74 |
|  | Replicate 3 | 40,376,742 | 34,665,246 | 3,779,860 | 85.85 |
