# Supplementary material for: Cohen Syndrome Patient iPSC-Derived Neurospheres and Forebrain-Like Glutamatergic Neurons Reveal Reduced Proliferation of Neural Progenitor Cells and Altered Expression of Synapse Genes
Source: J Clin Med. 2020 Jun 16;9(6):1886. doi: 10.3390/jcm9061886 (PMC7356975; doi:10.3390/jcm9061886)
Supplement: Supplementary file 1 [file jcm-09-01886-s001.zip › Supplementary headers and footers.docx]

**Supplementary Materials**

**Figure S1.** Generation of control iPSCs and neuronal differentiation from control iPSCs

**(a)** Morphology of iPSC colonies and alkaline phosphatase staining in iPSCs. Scale bar, 200 μm. **(b)** Immunostaining of control iPSCs with specific antibodies against intracellular pluripotent stem cell markers (Oct3/4, SSEA3, SSEA4, Tra1-60, and Tra1-81). Scale bar, 50 μm. **(c)** Normal karyotype in control iPSCs. **(d)** Immunocytochemical analysis of CS neurons differentiated from CS iPSCs using a glutamatergic marker, vGLUT1, a GABAergic marker, GAD67, and a neuronal marker, Tuj1 or MAP2. Scale bar, 20 μm. **(e)** Genomic sequence of the *VPS13B* gene in control fibroblasts, control iPSCs, and iPSC-derived neurons.

**Figure S2.** Rapid neuronal induction efficiency from control and CS iPSCs by expression of NGN2

**(a-c)** Quantification of rapid neuronal induction efficiency in control, CS1, or CS2 induced neurons differentiated from each iPSC. Bar graph represents mean ± SEM. One-way AVOVA, Turkey post-hoc test, ns, no significance.

**Figure S3.** Basal electrophysiological properties of CS iPSC-derived glutamatergic neurons

**(a-c)** Basic membrane properties of control and CS neuronal cells. No differences in **(a)** resting membrane potential, **(b)** capacitance, and **(c)** input resistance between control and CS neuronal cells. Both **(d)** sodium channel current measured by ramp voltage step and **(e)** potassium channel current were not altered in CS neuronal cells as compared to control neuronal cells. **(f)** Sodium and potassium channel currents induced by voltage steps (from -60mV to +50mV with 10mV increments) were not different between control and CS neuronal cells. **(g)** The number of action potentials induced by step current injection of CS neuronal cells was similar to control neuronal cells. **(h-j)** Action potential properties of control and CS neuronal cells. No differences were observed between **(h)** AP threshold, **(i)** AP amplitude, and **(j)** AP half-width between control and CS neuronal cells. Four independent cultures were used.

Figure S4. Visualizations of metascape results based on upregulated and downregulated gene lists. Heatmap showing the top enrichment clusters, one row per cluster, using a discrete color scale to represent statistical significance.

**Table S1.** Information of real-time PCR primers

**Table S2.** RNA-Seq read mapping summary

**Table S3.** A list of differentially expressed genes between iPSC-derived CS neurons and control neurons

**Table S4.** A list of GO terms enriched in upregulated and downregulated genes
