# Supplementary figures and images for: Cohen Syndrome Patient iPSC-Derived Neurospheres and Forebrain-Like Glutamatergic Neurons Reveal Reduced Proliferation of Neural Progenitor Cells and Altered Expression of Synapse Genes
Source: J Clin Med. 2020 Jun 16;9(6):1886. doi: 10.3390/jcm9061886 (PMC7356975; doi:10.3390/jcm9061886)

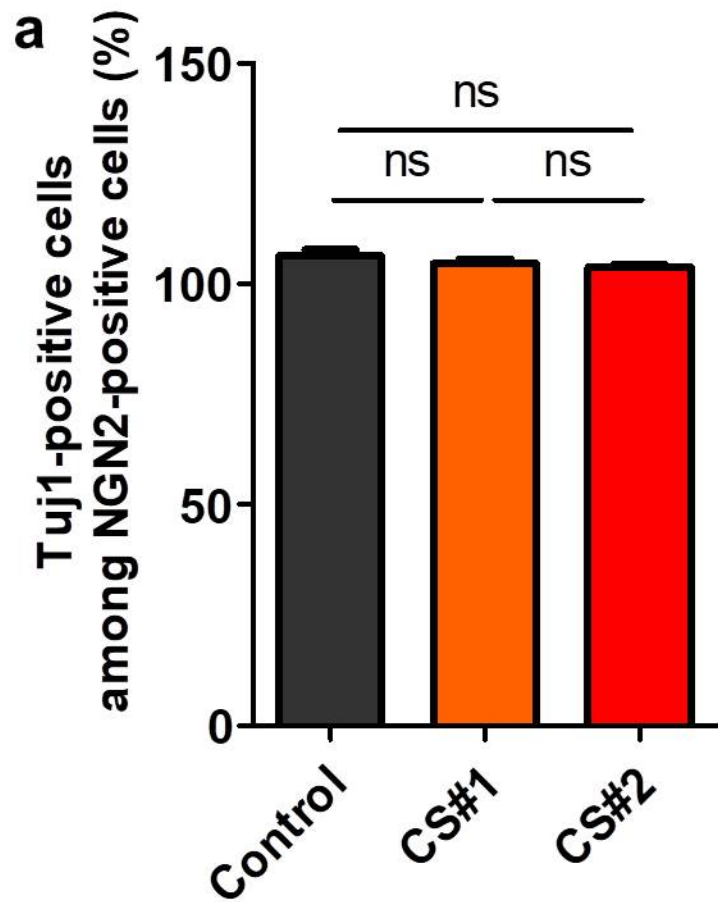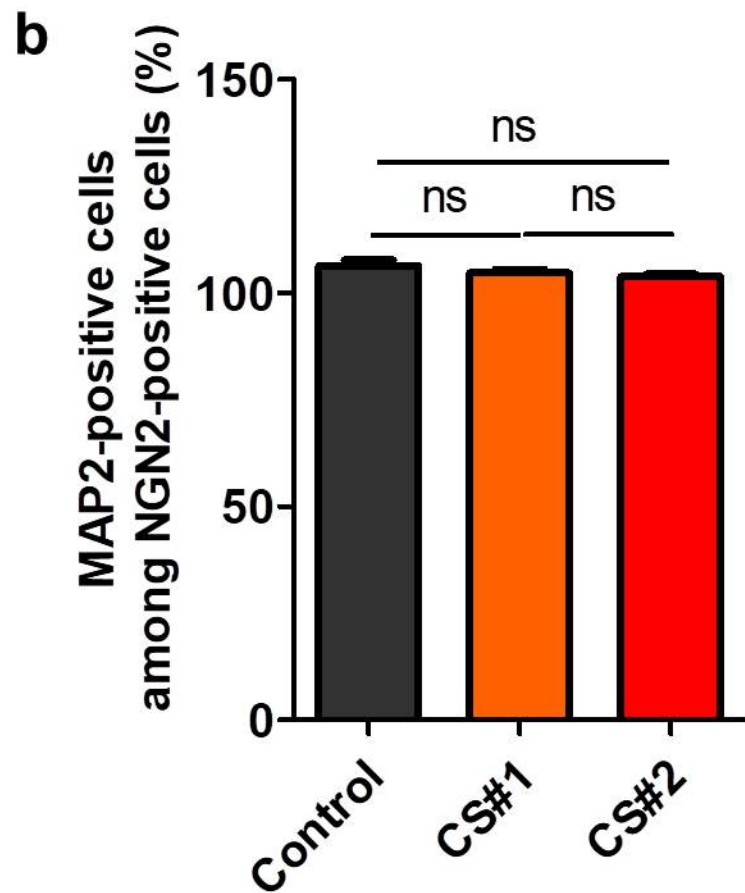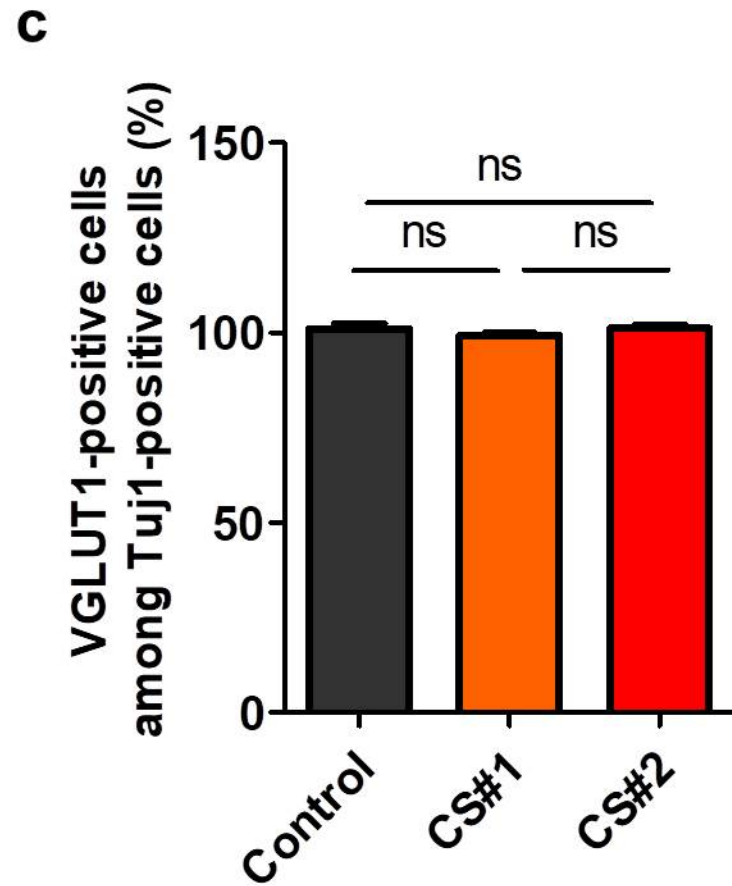

Supplement: Supplementary file 1 [file jcm-09-01886-s001.zip › Figure S2.pdf]

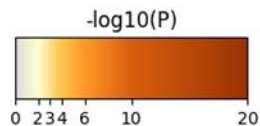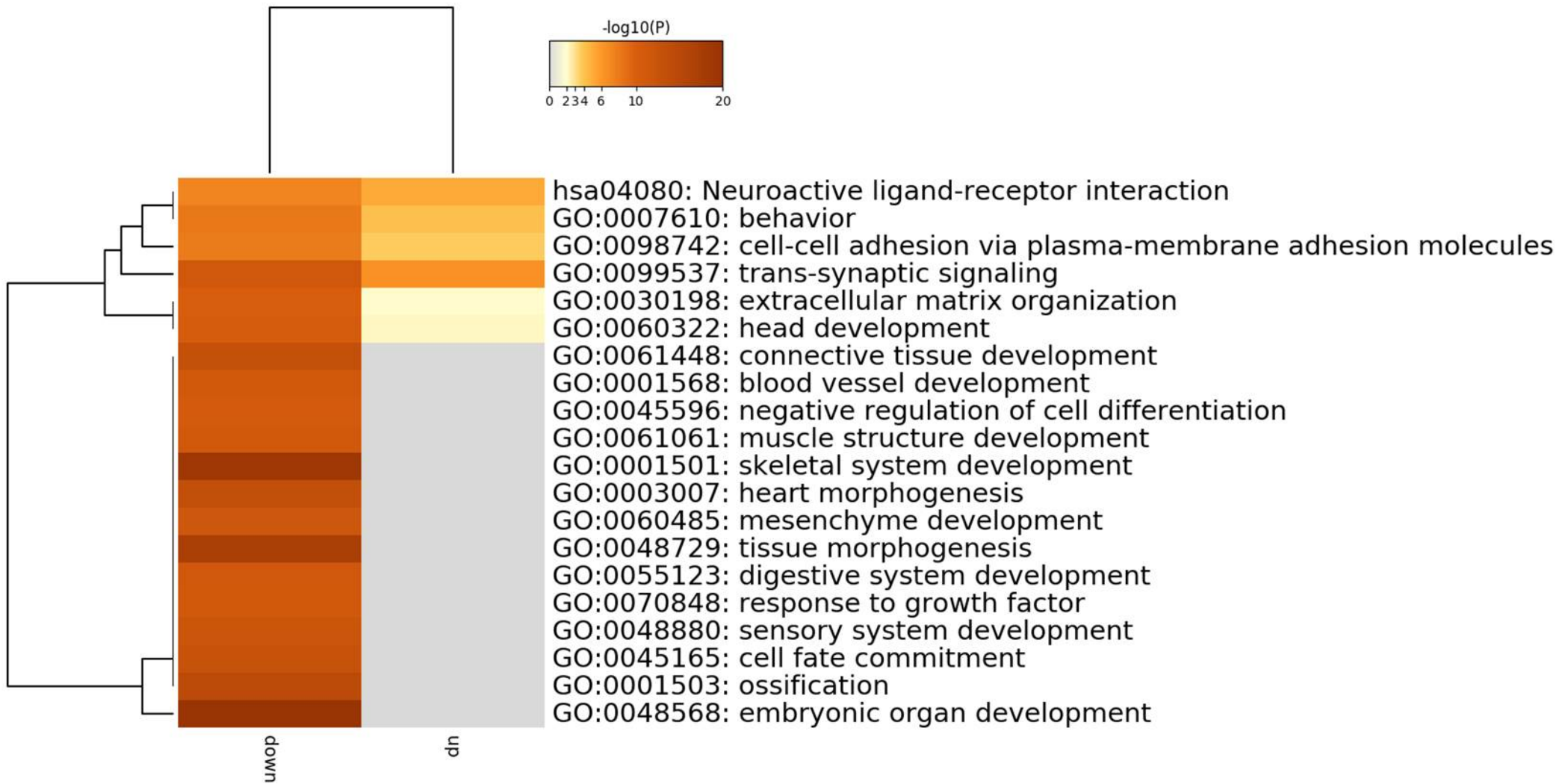

Supplement: Supplementary file 1 [file jcm-09-01886-s001.zip › Figure S4.pdf]

**a**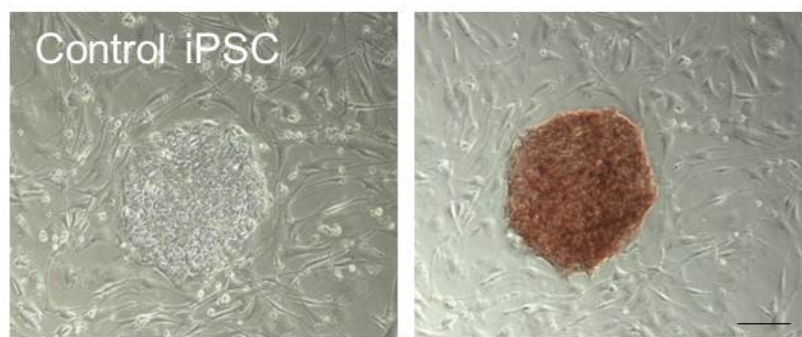**b**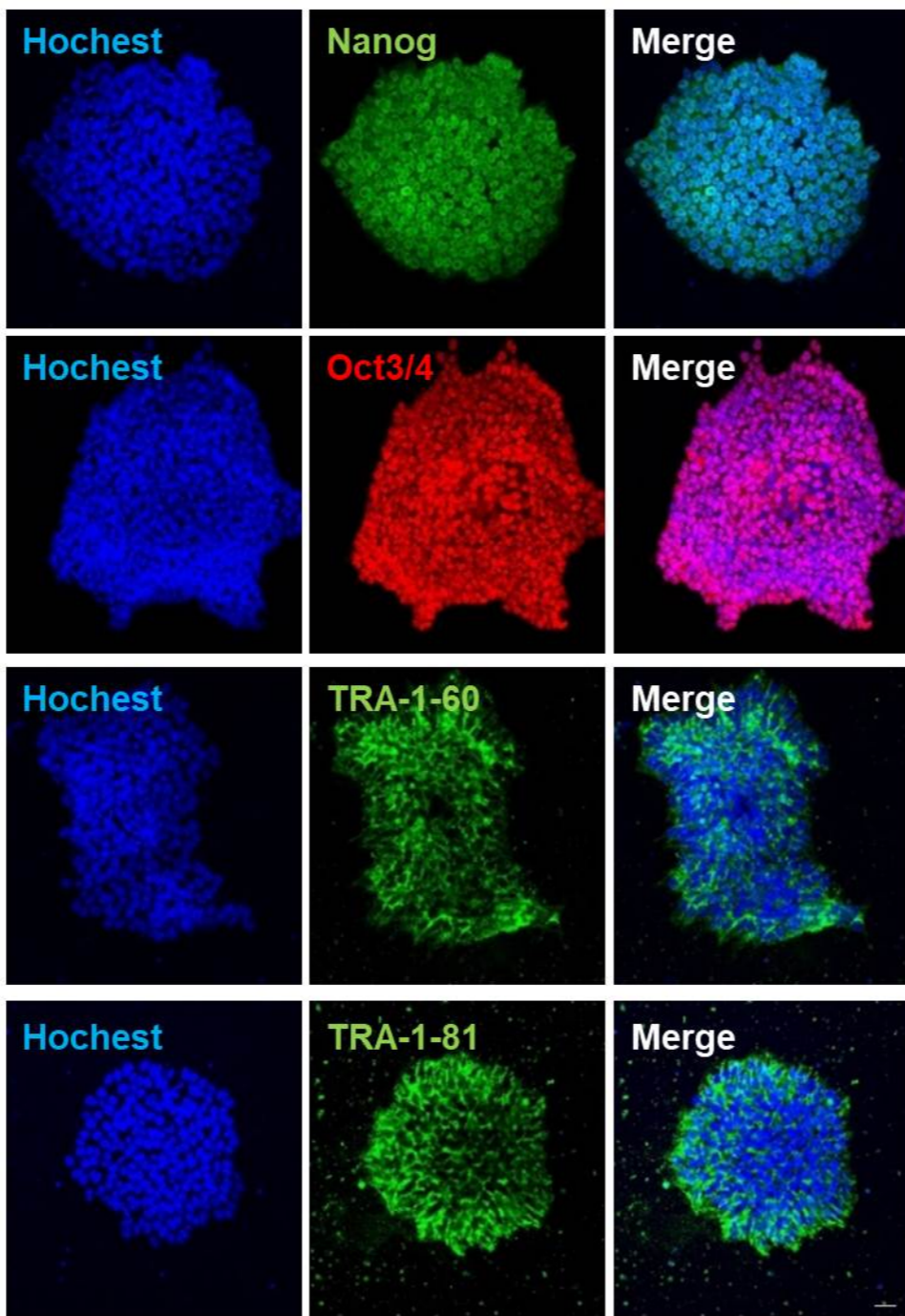**d**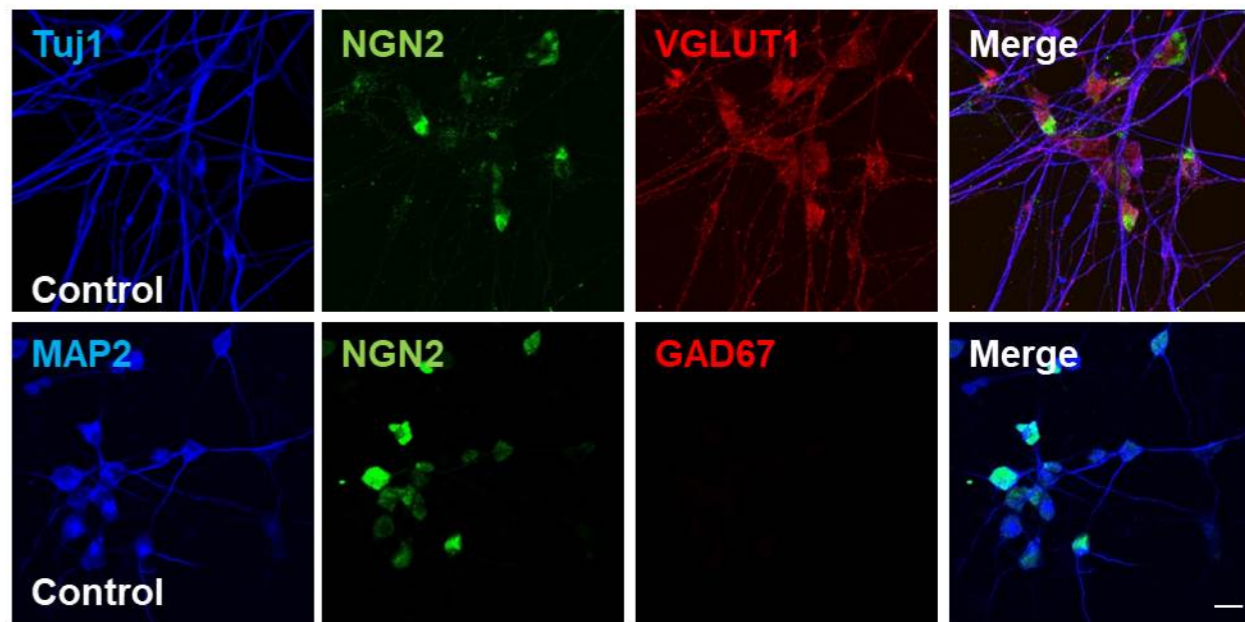**c****Control iPSC**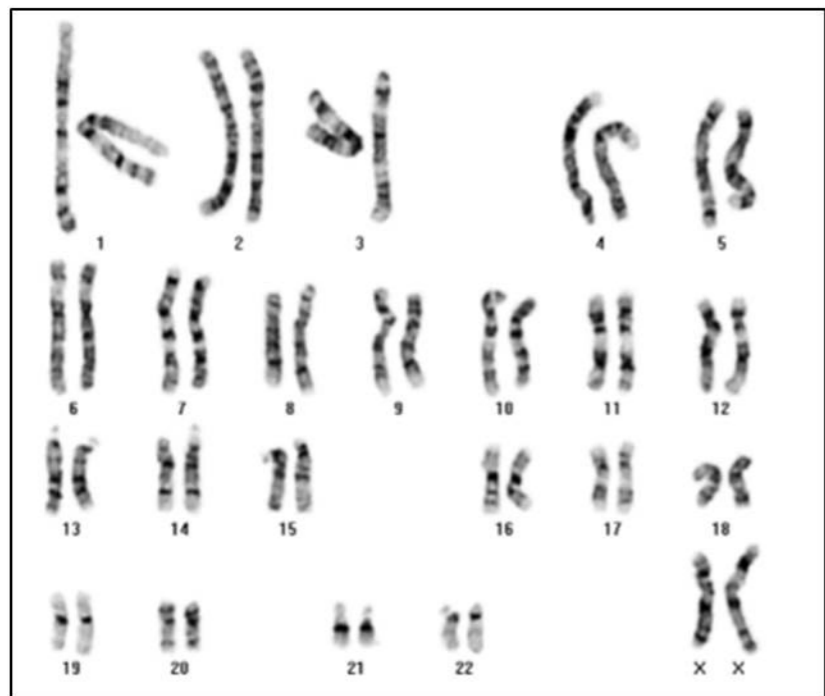**e**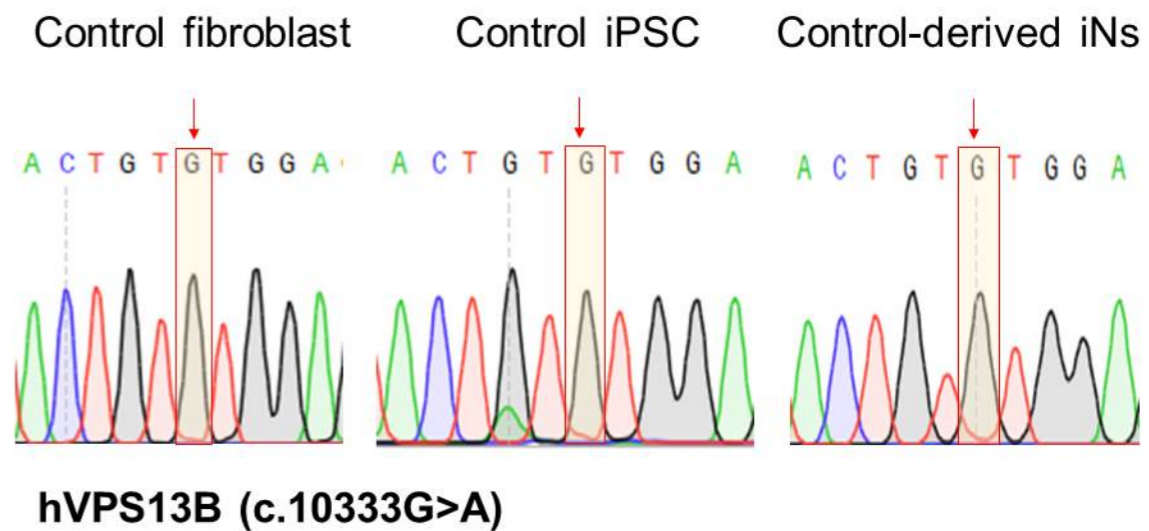

Supplement: Supplementary file 1 [file jcm-09-01886-s001.zip › Figure S1.pdf]
